# Thermographic Fault Diagnosis of Ventilation in BLDC Motors

**DOI:** 10.3390/s21217245

**Published:** 2021-10-30

**Authors:** Adam Glowacz

**Affiliations:** Department of Automatic Control and Robotics, Faculty of Electrical Engineering, Automatics, Computer Science and Biomedical Engineering, AGH University of Science and Technology, aleja Adama Mickiewicza 30, 30-059 Kraków, Poland; adglow@agh.edu.pl

**Keywords:** thermographic, infrared, BLDC motor, image processing, fault diagnosis

## Abstract

Thermographic fault diagnosis of ventilation in BLDC (brushless DC) motors is described. The following states of BLDC motors were analyzed: a healthy BLDC motor running at 1450 rpm, a healthy BLDC motor at 2100 rpm, blocked ventilation of the BLDC motor at 1450 rpm, blocked ventilation of the BLDC motor at 2100 rpm, healthy clipper, and blocked ventilation of the clipper. A feature extraction method called the Common Part of Arithmetic Mean of Thermographic Images (CPoAMoTI) was proposed. Test thermal images were analyzed successfully. The developed method, CPoAMoTI is useful for industry and society. Electric cars, trains, fans, clippers, computers, cordless power tools can be diagnosed using the developed method.

## 1. Introduction

The global electric motor manufacturing market was worth USD 163 billion in 2020. BLDC motors (brushless DC) are part of the market and they are used in different equipment such as cars, fans, clippers, computers, cordless power tools. They are used in the mining, computer, and car industry.

Financial losses are caused by unexpected faults in motors. Diagnosis systems can decrease the number of unexpected faults, which highlights the need to develop new diagnostic methods for electrical motors. Faults in electric motors can be electrical and mechanical. Electrical faults are often due to shorted coils and broken rotor bars in electrical circuits. Mechanical faults include ventilation, bearings, gear, and shaft faults. Faults are detected using thermal, current, visual, acoustic, vibration signals.

Fault diagnosis based on thermal imaging has not been explored in many scientific papers, which provides the motivation to develop thermographic fault diagnosis. This paper describes thermographic fault diagnosis of the ventilation in BLDC motors. A feature extraction method called the Common Part of Arithmetic Mean of Thermographic Images (CPoAMoTI) is proposed.

This article consists of 7 sections: (1) the introduction, (2) analyzed states of BLDC motors, (3) thermographic measurements for the BLDC, (4) the developed thermal fault diagnosis method, (5) results of the analysis, (6) discussion, and (7) conclusions.

### Related Work

Methods of fault diagnosis in motors has been developed by many scholars. The fault diagnosis of electrical faults is based on different diagnostic signals including thermal, acoustic, vibration, and electric current signals. Many proposed fault diagnosis methods are based on feature extraction and classification. Another approach is deep learning, for example, recursive, recurrent, and convolutional neural networks. Feature extraction, classification, and deep learning are often used for fault detection. Recently, several new methods of fault diagnosis have been developed, including thermal imaging, pyrometric analysis using infrared laser pyrometer, acoustic analysis, and vibration analysis, chemical and oil analysis [1,2,3,4,5,6,7,8,9,10,11,12,13,14,15,16,17,18,19,20,21].

Acoustic analysis is used for detecting different faults in motors, engines, fans, gearboxes. The advantages of acoustic analysis are non-invasive measurement, a short time is required for acoustic measurement, and good results are achieved, although a few training and test samples of the acoustic signal are required for proper recognition. The disadvantages of acoustic analysis are that some faults do not generate acoustic signals, and the noise of other signals interferes with the acoustic signal (reflected). Acoustic-based fault diagnosis has been developed in a number of studies in the literature [2,3,4,5,6].

Yao et al. presented a method for the acoustic fault diagnosis of a planetary gearbox that used the Fourier decomposition method and the random forest classifier. The fault diagnosis accuracy rate was equal to 96.32% [2]. Acoustic fault diagnosis for rotary-machine bearing faults is also presented in the literature. The proposed method was based on the spectrogram and convolutional neural network. This method recognized degradation levels and types of bearing faults. It achieved accuracy in the range of 93.33–98.21% [3]. An acoustic-based fault diagnosis method that uses acoustic images of bearings and a convolutional neural network was also proposed. This approach obtained an accuracy equal to 99.13% [4]. In another study, a fault identification method for a synchronous hydraulic motor was investigated. Four states of the synchronous hydraulic motor were analyzed. The authors of the paper used wavelet packet energy, the Pearson correlation coefficient, and the nearest neighbor classifier. The recognition accuracy was equal to 100% [5]. Additionally, acoustic fault diagnosis in three-phase induction motors was studied based on the SMOFS-20-EXPANDED method and LDA, NBC, CT. The total recognition efficiency was equal to 94.99% [6].

Vibration analysis is also used for detecting different faults in motors and gears. The vibration signal is not noisy (lack of reflected, interfered vibration signals) and it is easy to process. However, it is difficult to detect the localization of faults using vibration analysis. For this reason, methods based on a fusion of vibration and acoustic signals have been developed [7,8,9,10,11,12].

Vibration and acoustic fault diagnosis of gears are presented in the literature. In one study, a convolutional neural network using vibration and acoustic signals was used for the analysis. The accuracy of the proposed approach was equal to 97.7% [7]. Vibration and acoustic fault diagnosis of a gearbox are also described in another study where wavelet packet decomposition and wavelet denoising were used for pre-denoising. FFT and infinite impulse response filtering were used for feature extraction. Next, classification was carried out using a self-organizing map. This method achieved an accuracy equal to 95% for four different features [8]. A vibration-based technique for fault diagnosis of a gearbox that uses frequency-modulated empirical mode decomposition and support vector machine has also been described in the literature. The obtained accuracy was equal to 90% [9]. A vibration-based method for bearing fault diagnosis using a convolutional neural network and a deep forest model has been developed. The accuracy of the vibration-based method was equal to 99.4% [10]. Techniques for detecting tool breakage using vibration signals were demonstrated in [11]. Vibro-acoustic analysis of a railway disc brake based on the amplitude and frequency domains was conducted in [12].

Thermal imaging is often used for different applications such as fault diagnosis, medical diagnosis, security, car monitoring, plane monitoring, astronomy, and by rescue teams. Fault diagnosis based on thermal imaging is non-contact and non-invasive. It is a highly effective method of fault diagnosis [13,14,15,16,17,18,19,20,21]. However, the high cost of thermal imaging cameras is a limitation for fault diagnosis applications.

Thermal condition monitoring of bearing faults in three-phase induction motors has been proposed previously. Acquired thermal images were converted using the HSV model. Segmentation of the hue component was conducted using Prewitt, Roberts, Sobel, Canny filters. The computed results were good [13]. A convolutional neural network, K-means clustering, and support vector machine were used for three-phase induction motors. The accuracy was equal to 100% for real thermal images of the three-phase induction motor [14]. Thermal-based fault diagnosis of an induction motor been described in which an adaptive neuro-fuzzy inference system was used for faults such as air gap eccentricity, cooling fan failure, and shaft bending. The accuracy of the classification of thermal images was equal to 91.27% [15]. A fault diagnosis technique for an induction motor (working in transient conditions) based on thermal images and a convolutional neural network has been described. The accuracy of the proposed approach was equal to 100% for most cases [16]. Morales-Perez et al. analyzed three specific regions of thermography images to detect bearing damage. The authors measured a difference of 1.8 °C between the faulty and healthy bearing of the induction motor [17]. Furthermore, bearing faults in induction motors were analyzed using infrared thermography. This approach was based on 2D-discrete wavelet transform, principal component analysis, support vector machine, complex decision tree, and linear discriminant analysis. The accuracy was between 80–100% for healthy, inner race defects, and outer race defects. The SVM classifier recognized the test data set with high accuracy [18]. The thermal analysis of brushless direct current motor using thermal images is also presented in the literature. The following parameters were found for motors with corroded permanent magnets (reduction of 0.15 mm): reduced efficiency by 8% and reduced rated speed by 70 rpm [19]. A review of the industrial applications of infrared thermography is presented in [20,21].

## 2. Analyzed States of BLDC Motors

**Figure 1:**
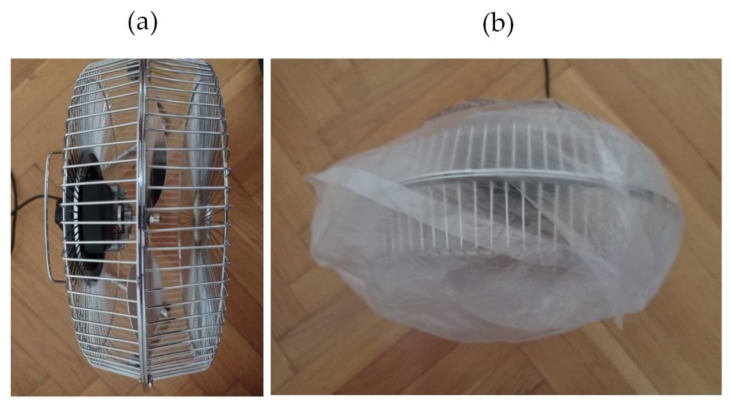
Analyzed fan: (**a**) healthy BLDC motor, (**b**) blocked ventilation of the BLDC motor.

**Figure 2:**
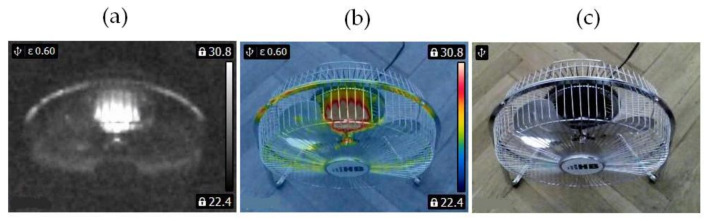
Healthy BLDC motor at 2100 rpm (**a**) gray-scale, (**b**) rainbow scale (**c**) photo.

**Figure 3:**
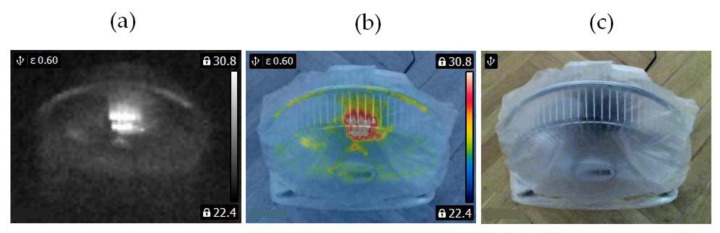
Blocked ventilation of the BLDC at 2100 rpm (**a**) gray-scale, (**b**) rainbow scale (**c**) photo.

**Figure 4:**
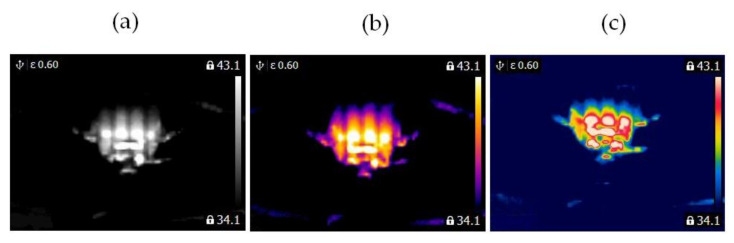
Healthy BLDC motor at 1450 rpm (**a**) gray-scale, (**b**) iron scale, (**c**) rainbow scale.

**Figure 5:**
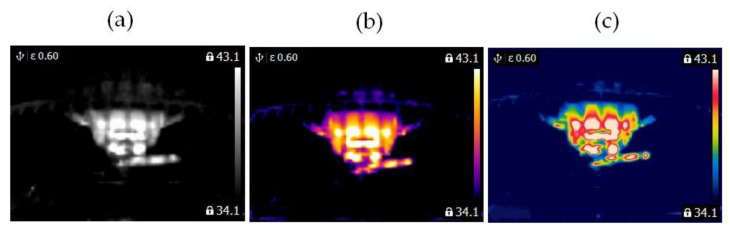
Blocked ventilation of the BLDC motor at 1450 rpm (**a**) gray-scale, (**b**) iron scale, (**c**) rainbow scale.

**Figure 6:**
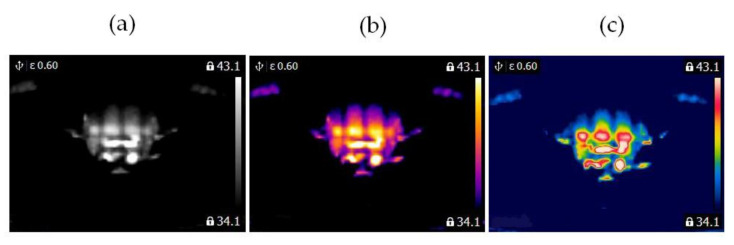
Healthy BLDC motor at 2100 rpm (**a**) gray-scale, (**b**) iron scale, (**c**) rainbow scale.

**Figure 7:**
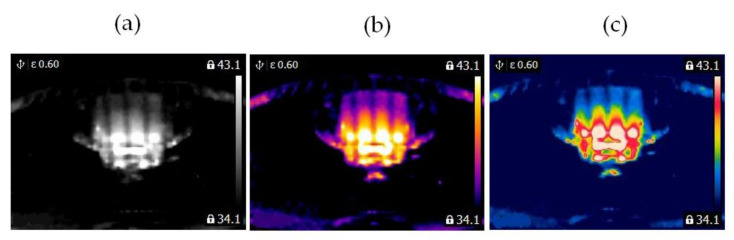
Blocked ventilation of the BLDC motor at 2100 rpm (**a**) gray-scale, (**b**) iron scale, (**c**) rainbow scale.

In this study, two similar fans (HB Polska Sp. z o. o., AirCoolic, model: AC 2320S, made in China) were analyzed (Figure 1, Figure 2 and Figure 3). Each fan consisted of a BLDC motor (25 W), steel blades and a cage. The fan was operated at different speeds. The following states of the BLDC motor were analyzed: healthy BLDC motor at 1450 rpm (Figure 4), blocked ventilation of the BLDC motor at 1450 rpm (Figure 5), healthy BLDC motor at 2100 rpm (Figure 6), and blocked ventilation of the BLDC motor at 2100 rpm (Figure 7).

The blade of the fan (Figure 3) obscured a part of the BLDC motor. Camera positioning is very important for measurements (Figure 4, Figure 5, Figure 6 and Figure 7). Thermal images of the BLDC motor (fan) are shown in Figure 4, Figure 5, Figure 6 and Figure 7.

**Figure 8:**
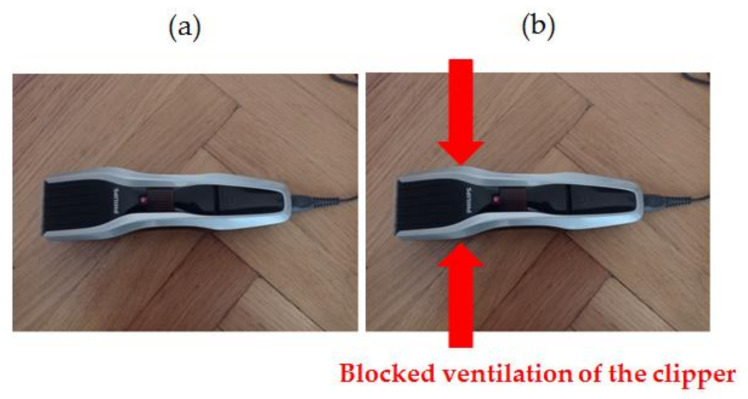
Analyzed clipper HC5440/80: (**a**) healthy clipper, (**b**) blocked ventilation of the clipper.

**Figure 9:**
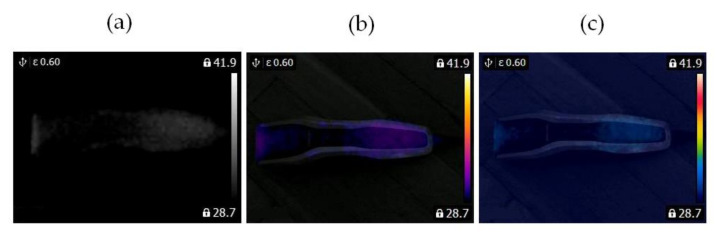
Healthy clipper: (**a**) gray-scale, (**b**) iron scale, (**c**) rainbow scale.

**Figure 10:**
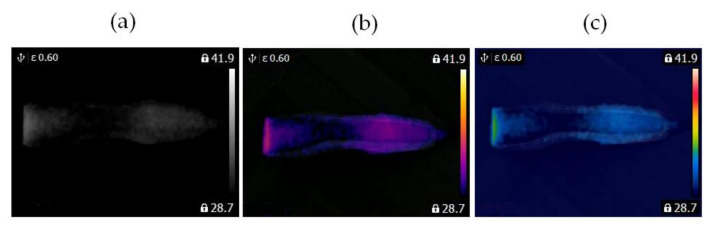
Blocked ventilation of the clipper: (**a**) gray-scale, (**b**) iron scale, (**c**) rainbow scale.

Two similar clippers (Clipper HC5440/80) were analyzed (Figure 8). Each clipper consisted of a BLDC motor (5.4 W). The following states of the clipper were analyzed: healthy clipper (Figure 8 and Figure 9), and blocked ventilation of the clipper (Figure 8 and Figure 10).

Thermal images of the BLDC motor (Clipper HC5440/80) are shown in Figure 9 and Figure 10.

## 3. Thermographic Measurements for the BLDC

**Figure 11:**
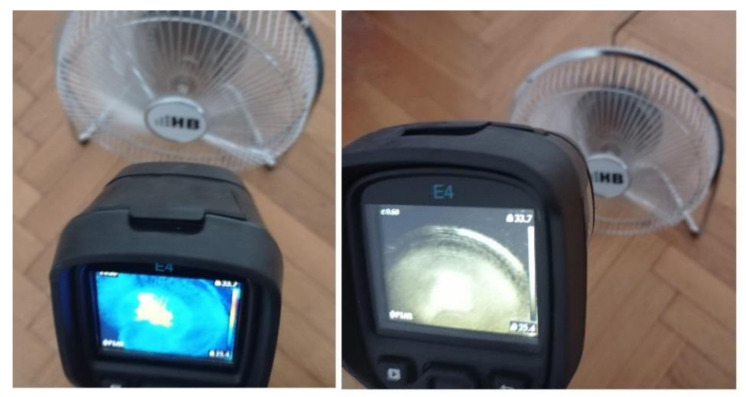
Thermographic measurements for the BLDC motor (fan).

**Figure 12:**
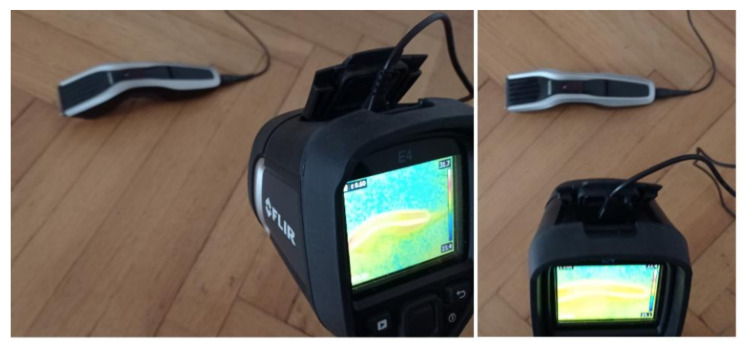
Thermographic measurements for the BLDC motor (clipper HC5440/80).

Thermographic measurements were carried out in a room (4 m × 5 m). The BLDC motor was at a distance of 0.2 m from the thermal imaging camera (Figure 11 and Figure 12). The BLDC motor of the fan has the following parameters: *PO_BLDC_*_1_ = 25 W (power of the BLDC), *RAS_BLDC_*_1_ = 1450 rpm, *RAS_BLDC_*_1_ = 2100 rpm (rotor angular speed), *VO_BLDC_* = 230 V/50 Hz (voltage), *WE_FAN_* = 1.6 kg (weight of the fan). The BLDC motor of the clipper has *PO_BLDC_*_2_ = 5.4 W (power of the BLDC). The parameters of the FLIR E4 camera are as follows: temperature measuring range (−20 °C to +250 °C), resolution of images (80 × 60), its thermal sensitivity is less than 0.15 °C, and the image frequency is equal to 9 Hz. Images of 80 × 60 pixels were converted to images of 320 × 240 pixels using FLIR software. The thermal camera was vibrating in the range of 0–0.5 m/s^2^. Different measured thermal images were used. A movie was recorded for two devices (two fans, two clippers). The thermal imaging camera was set at the highest temperature of the analyzed state, that is, blocked ventilation of the BLDC motor at 2100 rpm. The measurements were performed after a steady state was reached so as to avoid the negative effects of transients in the damage detection method. Measurements were carried out for 30 s of operation of the motor. The parameter ε (emissivity coefficient) was equal to 0.6. The parameter ε is between 0.62–0.73 for various types of steel. Rolled sheet steel (temperature 21 °C) has an emissivity coefficient equal to 0.660. Stainless steel 303, after 42 h of heating at 527 °C (temperature 216–527 °C) has an emissivity coefficient equal of 0.620−0.730 [22]. The author selected ε = 0.6 and it was good enough for measurements. Nylon covering was used for the detection of fan faults. The nylon blocking the ventilation leads to a temperature increase (indicative of damage for many conditions), but it does not affect the thermal imaging equipment thanks to its transparency. Nylon covering was used to simulate damage, thus allowing us to implement damage detection.

## 4. The Developed Thermal Fault Diagnosis Method

**Figure 13:**
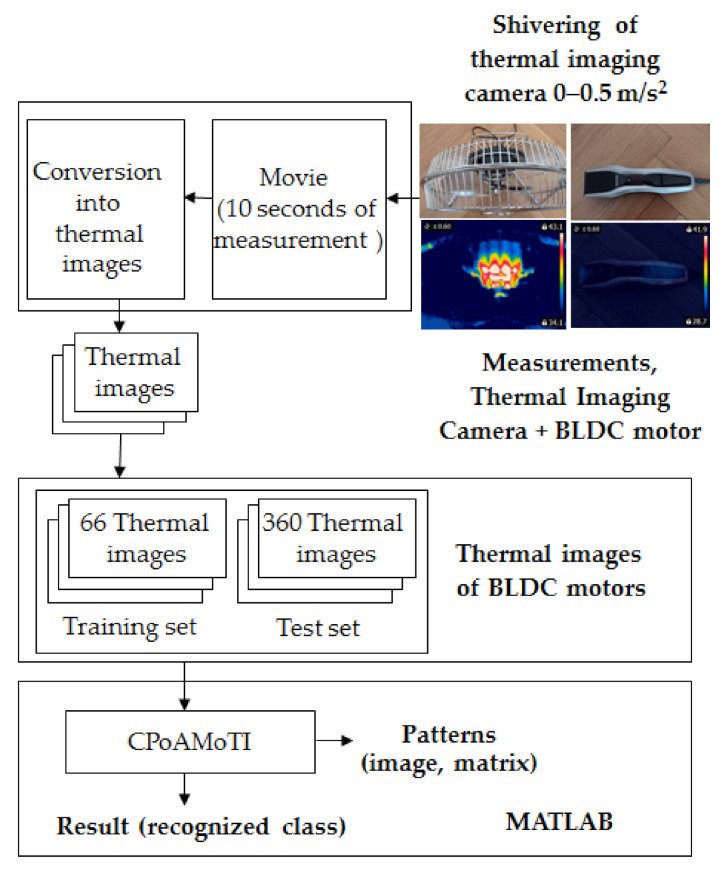
Block diagram of the developed thermal fault diagnosis technique using CPoAMoTI.

The developed thermal fault diagnosis technique using the Common Part of Arithmetic Mean of Thermographic Images (CPoAMoTI) is presented in Figure 13. Measurements were carried out using a thermal imaging camera FLIR E4 and a computer. The gray-scale movie was captured and then the movie was converted into gray-scale thermal images (format TIFF, 256 colors). Then, the CPoAMoTI method was used to compute patterns. It was used for training and test sets (66 and 360 gray-scale thermal images—6 classes for two BLDC motors). Next, image subtraction was carried out and differences in the images (test and training) were computed. Next, the sum of the pixels of the computed differences was calculated. The differences in the images determine the recognized class.

### 4.1. Common Part of Arithmetic Mean of Thermographic Images (CPoAMoTI)

The author developed a feature extraction method—CPoAMoTI. For a better understanding, it is demonstrated for the gray-scale thermal images of the analyzed fan (4 classes: 44 training and 240 test thermal images). The CPoAMoTI method includes the following steps:

1. Gray-scale thermal images (256 colors, matrices 320 × 240) are grouped as training and test sets.
2. Compute image of the arithmetic mean using thermal images of training set:
  (1) $$class_{k}=\frac{\sum_{n=1}^{n}\left|X_{n}\right|}{n_{}},$$
  where **X_n_** is the matrix of training thermal image, *n* is the number of training thermal images (*n* = 11), **class_k_** is the arithmetic means of class with **k** index (matrix 320 × 240), **class_1_** is the image of the arithmetic mean of training thermal images of the healthy BLDC motor at 1450 rpm, **class_2_** is the image of the arithmetic mean of training thermal images of the healthy BLDC motor at 2100 rpm, **class_3_** is the image of the arithmetic mean of the training thermal images of blocked ventilation of the BLDC motor at 1450 rpm, **class_4_** is the image of the arithmetic mean of the training thermal images of blocked ventilation of the BLDC motor at 2100 rpm (4 classes for the analyzed fan).
3. Compute differences:
  **diff*_j_*** = |**class_k_** − **class_g_**|, (2)
  where, **diff*_j_*** is the difference of two matrices; ***j*** is the number of computed differences for 4 classes ***j*** = 6, **diff_1_** = |**class_1_** − **class_2_**|,…, **diff_6_** = |**class_3_** − **class_4_**|; **k**, **g** is the number of classes, for 4 classes: 1, 2, 3, 4.
4. Compute the following sum:
  (3) $$sum\_avg=\sum_{j=1}^{j}\left|diff_{j}\right|,$$
5. Compute the value of *M*:
  *M* = max(**sum_avg**), (4)
  where *M* is the maximum value of matrix **sum_avg**.
6. Compute the value of *m*:
  *m* = *p* × *M*, (5)
  where *m* is the percentage of maximum value *M*, *p* is in the range of [0, 1]. The analysis is carried out for different parameters of *p*.
7. For each training and test thermal image, compute
  **C** = **TI** + **sum_avg** − **G**, (6)
  where **C** is the computed image; **G** is the matrix of 320 × 240, each element of matrix **G** has a value equal to *m*; **TI** is the training or test thermal image (matrix of 320 × 240).
8. In matrix **C**, set 0 for values less than zero. The computed images are as follows: images **C1**, **C2**, …, **C44** for the training set and **C51**, …, **C290** for the test set (only for analyzed fan).
9. Compute image subtraction:
  **d_i_** = |**C_a_** − **C_b_**|, (7)
  where, **d_i_** is the matrix of differences between test and training thermal images (for one test image and 44 training images, **d1**, **d2**, …, **d44** are computed), **C_a_** is the test thermal image (**C51**, …, **C290**), **C_b_** is the training thermal image (**C1**, **C2**, …, **C44**).
10. Compute sums of pixel values *s_i_* for each computed matrix **d_i_**,
  (8) $$s_{i}=\sum_{j=z}^{z}\left|pv_{z}\right|,$$
  where *s_i_* is the sum of pixel values for **d_i_**; *i* is the integer from 1 to 44; *pv_z_* is the pixel value, *z* is the integer from 1 to 76,800 (320 × 240 = 76,800).
11. Find the lowest value of the computed sums.
12. Detect the proper class of the BLDC motor.

**Figure 14:**
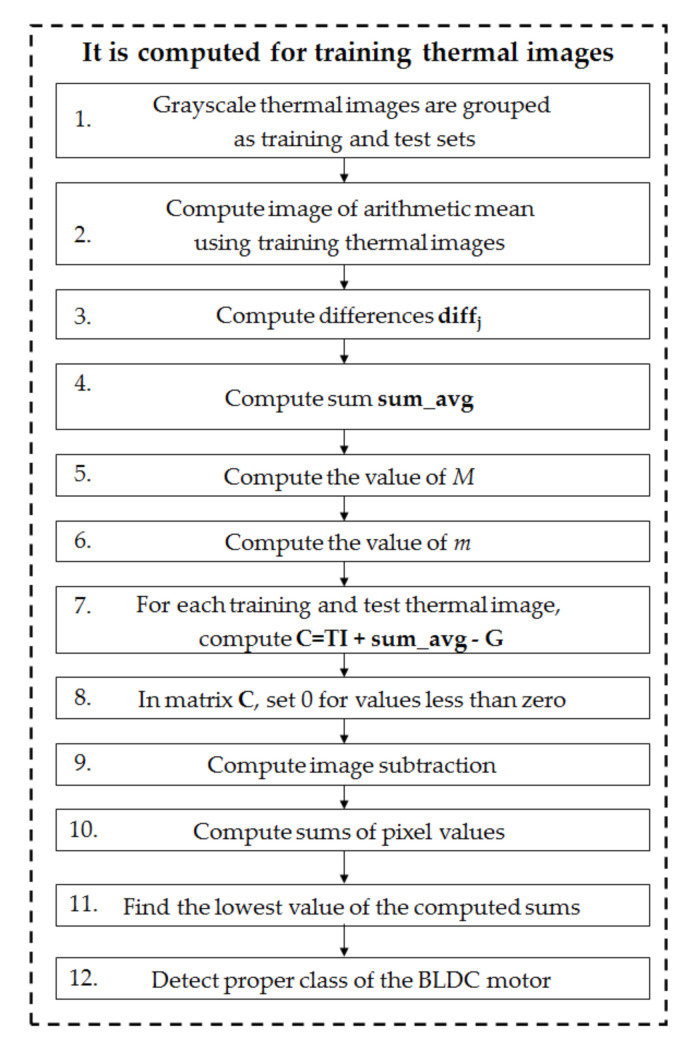
Flowchart of processing using the CPoAMoTI method.

A flowchart of the processing of training samples using the CPoAMoTI method is depicted in Figure 14.

**Figure 15:**
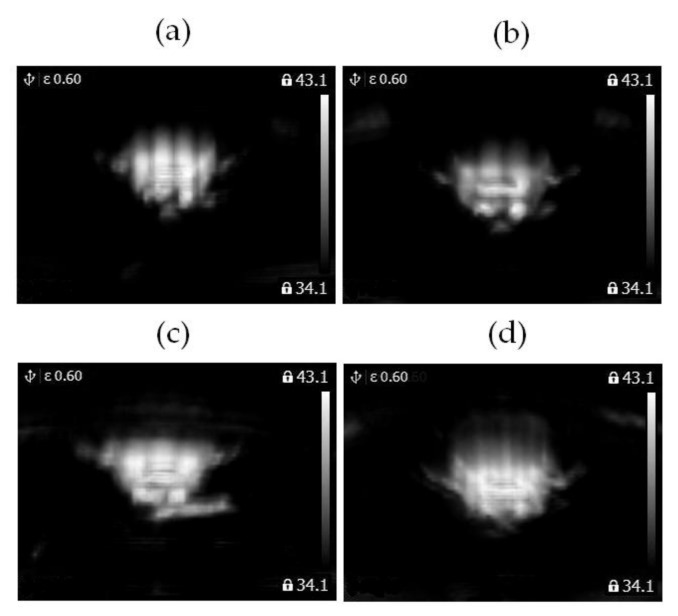
(**a**) Image of arithmetic mean of thermal images of the healthy BLDC motor at 1450 rpm (**class_1_**). (**b**) Image of arithmetic mean of thermal images of the healthy BLDC motor at 2100 rpm (**class_2_**). (**c**) Image of arithmetic mean of thermal images of the blocked ventilation of the BLDC motor at 1450 rpm (**class_3_**). (**d**) Image of arithmetic mean of thermal images of the blocked ventilation of the BLDC motor at 2100 rpm (**class_4_**).

Images of arithmetic means of 4 analyzed classes: **class_1_**, **class_2_**, **class_3_**, **class_4_** are presented in Figure 15.

**Figure 16:**
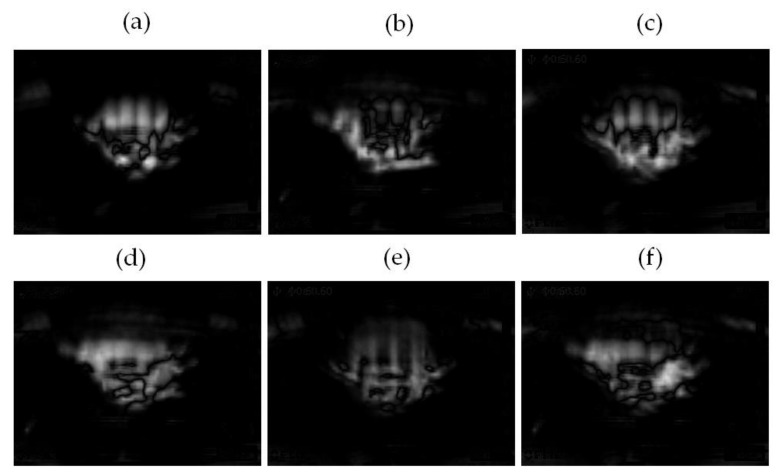
Computed difference: (**a**) **diff_1_** = |**class_1_** − **class_2_**|, (**b**) **diff_2_** = |**class_1_** − **class_3_**|, (**c**) **diff_3_** = |**class_1_** − **class_4_**|. Computed difference: (**d**) **diff_4_** = |**class_2_** − **class_3_**|, (**e**) **diff_5_** = |**class_2_** − **class_4_**|, (**f**) **diff_6_** = |**class_3_** − **class_4_**|.

The computed differences **diff_1_**, …, **diff_6_** are shown in Figure 16.

**Figure 17:**
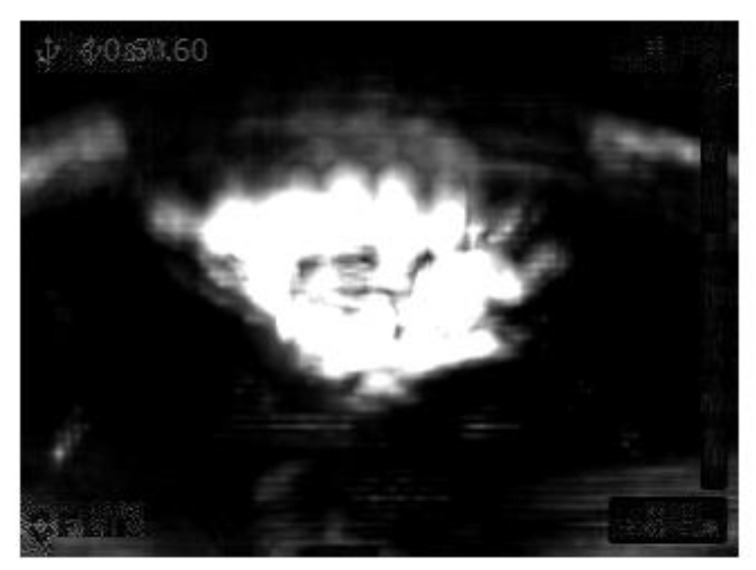
Computed matrix **sum_avg** (for the BLDC of the fan).

The obtained matrix **sum_avg** is presented in Figure 17.

**Figure 18:**
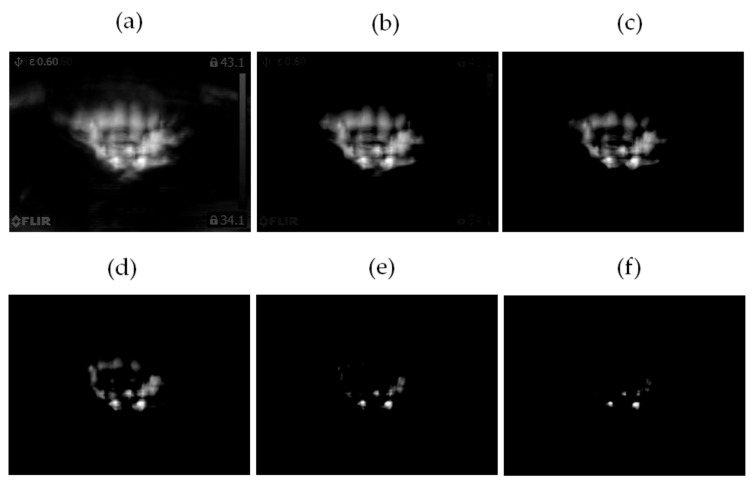
Thermal image of the healthy BLDC motor at 2100 rpm (**class_2_**) for the parameter (**a**) *p* = 0, (**b**) *p* = 0.3, (**c**) *p* = 0.5. Thermal image of the healthy BLDC motor at 2100 rpm (**class_2_**) for the parameter (**d**) *p* = 0.7, (**e**) *p* = 0.9, (**f**) *p* = 1.0.

The value of *M*, the maximum of matrix **sum_avg** is equal to 2.8784 for the considered training thermal images. This maximum is used for the equation *m* = *p* × *M*. The author analyzed the following *p* parameters: 0; 0.3; 0.5; 0.7; 0.9; 1.0. Next, step 7 was performed: **C** = **TI** + **sum_avg** − **G**. The thermal images of the healthy BLDC motor at 2100 rpm for different parameters *p* are shown in Figure 18.

**Figure 19:**
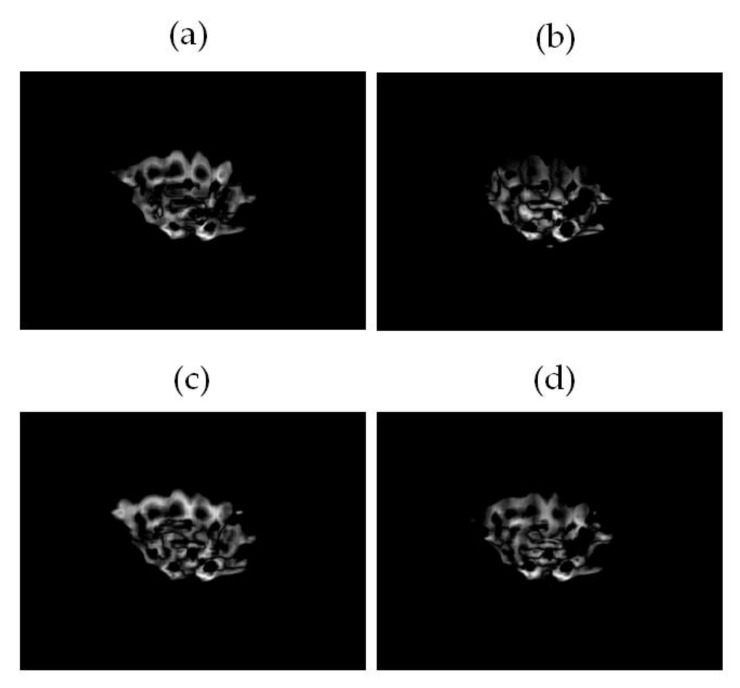
Computed difference **d_i_**: (**a**) |test_**class_2_** − training_**class_1_**|, (**b**) |test_**class_2_** − training_**class_2_**|. Computed difference **d_i_**: (**c**) |test_**class_2_** − training_**class_3_**|, (**d**) |test_**class_2_** − training_**class_4_**|.

Thermal images of the healthy BLDC motor at 2100 rpm for parameter *p* = 0.3–0.5 are the best recognized by the human eye (Figure 18b,c). Step 9 performs image subtraction. This step is similar to the KNN classifier [23,24]. Forty-four training thermal images were used (for fans). For each test thermal image, there are 44 differences. Four differences (of 44 computed differences, parameter *p* = 0.5) are presented in Figure 19.

Computed sums of the pixel values for 4 differences (parameter *p* = 0.5) are shown in Table 1). The class “healthy BLDC motor at 2100 rpm” is detected (sum of pixel values equals 975.5).

## 5. Results of the Analysis

The following states of the BLDC motor were analyzed: healthy BLDC motor at 1450 rpm, healthy BLDC motor at 2100 rpm, blocked ventilation of the BLDC motor at 1450 rpm, blocked ventilation of the BLDC motor at 2100 rpm, healthy clipper, and blocked ventilation of the clipper. To compute the common part of the arithmetic mean of thermographic images, the author used 11 training samples from each class (66 training samples in total). The author used 60 new test samples from each class (360 test samples in total) for testing. The cross-validation method was used for validation.

The recognition efficiency of one class *E_BLDC_* is expressed as Equation (9):

(9) $$E_{BLDC}=100\%\times (TTIRC)/(ATTI),$$

where *TTIRC* is the test thermographic images (for one class) recognized correctly, *ATTI* is all test thermographic images (for one class, 60 test images for the presented analysis).

The arithmetic mean of *E_BLDC_* (*AME_BLDC_*) is expressed as Equation (10):

(10) $$AME_{BLDC}=(E_{BLDC1}+E_{BLDC2}+E_{BLDC3}+E_{BLDC4})/4,$$

where *E_BLDC_*_1_ is the *E_BLDC_* for the healthy BLDC motor at 1450 rpm, *E_BLDC_*_2_ is the *E_BLDC_* for the healthy BLDC motor at 2100 rpm, *E_BLDC_*_3_ is the *E_BLDC_* for blocked ventilation of the BLDC motor at 1450 rpm, *E_BLDC_*_4_ is the *E_BLDC_* for blocked ventilation of the BLDC motor at 2100 rpm.

(11) $$AME_{BLDC}=(E_{BLDC5}+E_{BLDC6})/2,$$

where *E_BLDC_*_5_ is the *E_BLDC_* for the healthy clipper, *E_BLDC_*_6_ is the *E_BLDC_* for blocked ventilation of the clipper.

The results of the CPoAMoTI method for the BLDC motor (fan) are presented in Table 2 and Table 3.

The computed results (Table 2 and Table 3) are very good for the CPoAMoTI method for all analyzed states of the BLDC motor (fan) *AME_BLDC_* = 100% (parameter *p* in the range of 0–0.9). The thermographic images are very similar. However, the proposed method CPoAMoTI is good enough to correctly recognize four classes.

The results of the CPoAMoTI method for the BLDC motor (Clipper HC5440/80) is presented in Table 4.

The computed results (Table 4) are very good for the *CPoAMoTI* method. For all analyzed states of the BLDC motor (Clipper HC5440/80) *AME_BLDC_* = 100% (parameter *p* in the range of 0–1.0).

## 6. Discussion

BLDC motors are used in the mining, computer, and car industry. Thermographic fault diagnosis is non-invasive. All objects emit thermal radiation, which can be measured and analyzed. This study develops a new thermographic fault diagnostic method and was motivated by other research in this area [13,14,15,16,17,18,19,20,21]. Thermographic fault diagnosis of BLDC motors can also be used for other applications (other types of motors, combustion engines).

Measurements were carried out in a room with a temperature ranging from 28.7 to 34.1 °C. The thermal imaging camera sets the scale itself depending on the maximum temperature and background temperature. The proposed method CPoAMoTI has a high recognition rate. The presented analysis is based on pattern recognition. Many experimental parameters, for example, a distance of an object to the camera or orientation can be set. The motor can be dirty or painted and weather, ambient temperature, and humidity may vary. Similar to the human brain, we need a proper base of patterns and many thermal images for a variety of conditions.

Nylon covering was used for the detection of fan faults. Plastic or paper boxes can also be used. The analyzed fan did not have housing although a BLDC motor is usually in housing, for example, the fan of a computer or clipper.

The proposed feature extraction method CPoAMoTI is presented in Section 4.1. The proposed fault diagnosis works for measurements with and without vibration (Section 2, Section 3, Section 4 and Section 5). The results will always be good for measurements without vibrations. The computed training and test thermal images are almost the same. However, the results can be different for significant vibrations. In the proposed analysis, the vibrations of the thermal imaging camera were in the range of 0–0.5 m/s^2^. The computed results were quite good (*AME_BLDC_* = 100%) for the CPoAMoTI method. The author considered vibrations in the range of 0–0.5 m/s^2^ for the training and test sets. Both sets were different. Thresholding of the CPoAMoTI was carried out using Equation (6). Unnecessary elements in the image (labels, temperature, scale bar) were removed. The BLDC motor was at a distance of 0.2 m from the thermal imaging camera. The distance between the thermal imaging camera and the analyzed motor, and the vibration of the thermal imaging camera have an influence on the computed results.

Previous studies have developed thermographic fault diagnosis techniques for the three-phase induction motor (power of the motor = 550 W) [25] using the feature extraction MoASoID method, which used thermal images. This was based on the analysis of magenta color images (extracted from a color movie). The temperature of the analyzed thermal images was in the range of 21 to 38.7 °C. The measurement was carried out using the thermal imaging camera without vibrations. Thresholding of the MoASoID was carried out using binarization (one time). The MoASoID method had problems with unnecessary elements in the image (labels, temperature, scale bar). The MoASoID used the differences between the images of training and test sets. It computed one feature—the sum of the pixels. The method was tested for three classes: a healthy motor, a motor with two broken bars, and a motor with a faulty ring in the squirrel-cage. The recognition rate of the MoASoID was equal to 100% [25].

In another study, the author describes the thermographic fault diagnosis technique, which was used for an electric impact drill (commutator motor, power of the motor = 500 W). The feature extraction BCAoID method was presented. The BCAoID method used thermal images. It was based on the analysis of gray-scale images (extracted from the gray-scale movie). The temperature range of the analyzed thermal images was from 27.6 to 39 °C. The measurement was carried out using the thermal imaging camera with a 0.05 m offset between the training and test thermal images. Thresholding of the BCAoID was carried out using binarization (two times). Unnecessary elements in the image (labels, temperature, scale bar) were removed. The BCAoID used the differences between the images of the training and test sets. It computed one feature—the sum of pixels. The method was tested for 3 classes: a healthy electric impact drill (EID), EID with a faulty fan (10 broken fan blades), EID with damaged gear train. The recognition rate of the BCAoID was in the range of 97.91 to 100% [26].

All three methods (MoASoID, BCAoID, and CPoAMoTI) are compared in Table 5. It can be observed that the CPoAMoTI method is more reliable than the other methods.

## 7. Conclusions

Thermographic fault diagnosis of the ventilation in BLDC motors was described in this paper. The feature extraction method, the Common Part of Arithmetic Mean of Thermographic Images (CPoAMoTI) was proposed. The following states of BLDC motors were analyzed: a healthy BLDC motor at 1450 rpm, healthy BLDC motor at 2100 rpm, blocked ventilation of the BLDC motor at 1450 rpm, blocked ventilation of the BLDC motor at 2100 rpm, a healthy clipper, and blocked ventilation of the clipper. Test thermal images were analyzed successfully. The recognition rate was equal to 100% for the analyzed states of the BLDC motors. Similar faults in motors can be analyzed using the presented CPoAMoTI method. The proposed thermographic fault diagnosis is non-invasive and the developed CPoAMoTI method is beneficial for industry and society. Electric cars, trains, fans, clippers, computers, cordless power tools can be diagnosed using the developed method. The author will analyze different thermal imaging cameras, signals, and faults in machines in future research. Different sensors such as microphones, accelerometers, and cameras will be used for the diagnosis of various machines.

## Figures and Tables

**Table 1:** Computed sums of pixel values for 4 differences (parameter *p* = 0.5).

State of the BLDC (Fan)	Sum of Pixel Values
healthy BLDC motor at 1450 rpm	1575.6
healthy BLDC motor at 2100 rpm	975.5
blocked ventilation of the BLDC motor at 1450 rpm	1910.9
blocked ventilation of the BLDC motor at 2100 rpm	1428.3

**Table 2:** Results of recognition for CPoAMoTI method (parameter *p* = 1.0) for the BLDC motor (fan).

State of the BLDC	*E_BLDC_* [%]
*E_BLDC_*_1_, healthy BLDC motor at 1450 rpm	100
*E_BLDC_*_2_, healthy BLDC motor at 2100 rpm	98.33
*E_BLDC_*_3_, blocked ventilation of the BLDC motor at 1450 rpm	100
*E_BLDC_*_4_, blocked ventilation of the BLDC motor at 2100 rpm	100
	*AME_BLDC_* [%]
*AME_BLDC_*	99.58

**Table 3:** Results of recognition for CPoAMoTI method (parameter *p* = 0–0.9) for the BLDC motor (fan).

State of the BLDC	*E_BLDC_* [%]
*E_BLDC_*_1_, healthy BLDC motor at 1450 rpm	100
*E_BLDC_*_2_, healthy BLDC motor at 2100 rpm	100
*E_BLDC_*_3_, blocked ventilation of the BLDC motor at 1450 rpm	100
*E_BLDC_*_4_, blocked ventilation of the BLDC motor at 2100 rpm	100
	*AME_BLDC_* [%]
*AME_BLDC_*	100

**Table 4:** Results of recognition for CPoAMoTI method (parameter *p* = 0–1.0) for the BLDC motor (Clipper HC5440/80).

State of the BLDC	*E_BLDC_* [%]
*E_BLDC_*_5_, healthy clipper	100
*E_BLDC_*_6_, blocked ventilation of the clipper	100
	*AME_BLDC_* [%]
*AME_BLDC_*	100

**Table 5:** Comparison of analysis and methods: MoASoID, BCAoID and CPoAMoTI.

Analyzed Method	MoASoID	BCAoID	CPoAMoTI
Type of motor	Three-phase induction motor	Commutator motor	BLDC motor
Power of the analyzed motor	550 W	500 W	25 W, 5.4 W
Analyzed faults of the motor	electrical	mechanical	mechanical
Temperature range of analyzed thermal images	21–38.7 °C	27.6–39 °C	34.1–43.1 °C 28.7–41.9 °C
Measurement with Vibrations	No	0.05 m offset	Vibration 0–0.5 m/s^2^
Thresholding	Binarization, 1 time	Binarization, 2 times	**C** = **TI** + **sum_avg** − **G**, negative values to 0
Problems with unnecessary elements in the image (label, temperature, scale bar)	Yes	No	No
Differences	Between images of training and test sets	Between images of training and test sets	Between arithmetic means of training classes
Number of analyzed features	1 feature— Sum of pixels	1 feature— Sum of pixels	Matrix 320 × 240
Number of analyzed classes	3	3	4 + 2
Recognition	Nearest Neighbor classifier, K-means, backpropagation neural network)	Nearest Neighbor classifier and the backpropagation neural network	Difference between features (matrices **C**)
Scale	Rainbow	Gray-scale	Gray-scale
Recognition Rate (%)	100	97.91–100	100

## Data Availability

Not applicable.
